# Valuing the contribution of sport volunteering to subjective wellbeing: evidence from eight European countries

**DOI:** 10.3389/fspor.2024.1308065

**Published:** 2024-02-02

**Authors:** Themistocles Kokolakakis, Jelle Schoemaker, Fernando Lera-Lopez, Willem de Boer, Vilma Čingienė, Alma Papić, Gerd Ahlert

**Affiliations:** ^1^ Sport Industry Research Group, Sheffield Hallam University, Sheffield, United Kingdom; ^2^ Sports & Economics Research Centre, HAN University of Applied Sciences, Nijmegen, Netherlands; ^3^ Department of Economics, Public University of Navarra, Pamplona, Spain; ^4^ Faculty of Public Governance and Business, Institute of Management and Political Sciences, Mykolas Romeris University, Vilnius, Lithuania; ^5^ Croatian Olympic Committee, International Cooperation and Corporate Communications Department, Zagreb, Croatia; ^6^ GWS, Gesellschaft für Wirtschaftliche Strukturforschung mbH, Osnabrück, Germany

**Keywords:** volunteering, sport clubs, subjective wellbeing, Europe, monetization, monetary value, wellbeing valuation approach

## Abstract

**Introduction:**

Volunteering is a prominent and integral aspect of the activities undertaken by sports clubs in Europe. However, even with its growing importance, quantifying the monetary worth of this nonmarket activity, in terms of wellbeing, can present certain difficulties. Traditional approaches to valuing volunteering (i.e., replacement and opportunity cost approaches) do not fully capture the value of volunteering to individuals, as they do not consider the intangible benefits that individuals may derive from their participation.

**Methods:**

This research provides added value to the monetisation of volunteering in sport by applying the wellbeing valuation approach (WVA) for the first time to a cross-sectional data in eight European countries. A double instrumental variable approach was developed to correct for unobservable variables that may influence the pairs: income and subjective wellbeing (SWB), and volunteering and SWB. This allows to estimate the causal impact of volunteering and income on SWB more accurately and assign a reasonable monetary value to this non-market activity.

**Results:**

The results, based on a sample size of 1,091, show an income compensation for a volunteer, devoting on average 8.7 hours during a four-week period of €16 to €50 per hour, equivalent to between € 1,700 and € 5,200 per year, depending on the nationality of the volunteer.

**Discussion:**

With these estimations insights into the value of volunteering in sports are provided, contributing to a better understanding of how this activity can be valued and supported. By recognising and accurately valuing the contributions of volunteers, sport organizations and policymakers can develop more effective strategies for promoting and supporting volunteering in sports.

## 1 Introduction

The sports policy of most European countries mainly targets sport clubs (1) and volunteering has become a key resource for the provision of affordable sporting activities in them (2). In this way, sport volunteering has long been recognised as an important contributor to the development and sustainability of sports organizations and events (3, 4). Specifically, within the realm of formal sport volunteering in numerous countries (5), volunteering within sports clubs takes precedence. This is primarily because it typically entails a consistent commitment, whereas sport events often necessitate volunteers only during the event's preparation and execution. Volunteers provide essential support for sports clubs in areas such as coaching, officiating, event organization, and facility maintenance (6, 7). Besides the advantages volunteering offers to sports organisations and others (2, 5), it also holds the potential to enhance the wellbeing of individuals who engage in volunteer activities (8, 9). Numerous empirical investigations have examined the factors influencing participation in sport volunteering [e.g., (10–14)]. Dawson and Downward (15) discovered that the same determinants applied to the choices of both engaging in sports and volunteering in sports. Rowe (16) contended that these two activities are integral components of sporting capital. However, even though volunteering in sports holds significant importance, evaluating its monetary worth can be complex due to its nature as non-market goods and services (17, 18). In addition, there are philosophical concerns about attributing monetary values to outcomes and impacts in general as well as discomfort with summing a range of social values into a single financial value (19). Traditional approaches to valuing volunteering, such as the replacement cost approach or the opportunity cost approach, focus on the input of individuals rather than the outcomes in terms of obtained benefits (18, 20, 21). These approaches may not fully capture the value of volunteering to individuals, as they do not consider the intangible benefits that individuals may derive from their participation (22). To address this issue, researchers have begun to apply the wellbeing valuation approach (WVA) to volunteering in sports (22, 23). This approach estimates the contribution of volunteering to individuals’ subjective wellbeing (SWB) and assigns monetary values by estimating compensation payments (24, 25). In other words, it calculates how much income individuals would be willing to forego in order to continue volunteering while retaining their current level of wellbeing.

The application of the WVA to volunteering in sports is still in its early stages, and there is much to be learned about how this approach can be used to accurately capture the value of volunteering. This paper aims to contribute to this growing body of research by examining the monetary value of volunteering in sports using the WVA method. Through a review of existing literature and an analysis of data from a large-scale survey conducted in eight European countries, the relationship between volunteering in sports and SWB will be explored. In the analysis, a double instrumental variable approach was exploited to correct for unobservable variables that may influence income and SWB and volunteering and SWB, as suggested by Schoemaker (26). This allows to estimate the causal impact of volunteering on SWB more accurately and assign a monetary value to this activity. Previous studies did only correct for one of these estimates (between income and SWB) and therefore are subjected to potential endogeneity biases. Moreover, this is the first study that employed a cross-sectional design in eight different European Countries. The study's emphasis is on understanding the value of volunteering in sports, contributing to a broader comprehension of how such activities are perceived and valued across different societies. The current study is based on the dataset that was created by the research team of the project “Economic Dimension of Volunteering in Sport” (EVIS) co-funded by the Erasmus + Programme of the European Union. The sample analysed may become available upon application from SportsEconAustria Institute of Sport Economy, as the organisation that conducted the data collection.

The rest of the paper is structured as follows. Section 2 presents the framework for monetisation of volunteering; Section 3 describes the data employed and the variables used and provides justification for the causal effects under analysis; Section 4 shows the main results. Conclusions, policy implications and limitation of the paper are presented in Section 5.

## 2 Literature review

### 2.1 Volunteering in sports clubs and its monetary contribution

Volunteers are individuals who freely offer their time and skills to support non-profit organizations or causes without expectation of financial compensation and where the members of the organisation are the main beneficiaries of voluntary work (27). Volunteers play a crucial role in supporting sports organizations and events. They provide essential support in areas such as coaching, officiating, event organization, and facility maintenance (3, 6). Without the contributions of volunteers, many European sports clubs would struggle to operate effectively. Consequently, the European Union has developed some funding opportunities and programs to boost volunteering (28).

Empirical studies have highlighted the importance of volunteers in supporting the development and sustainability of sports organizations. For example, a study by Wicker and Breuer (8) examined the organizational capacity and problems of disability sports clubs in Germany. The study found that volunteers played a crucial role in supporting the operations of these clubs, and that clubs with higher levels of volunteer involvement had greater organizational capacity. Misener and Doherty (29) conducted a study that examined the relationship between volunteer management practices and organizational capacity in community sports organizations. The study found that organizations with more effective volunteer management practices, such as providing training and recognition to volunteers, had higher levels of organizational capacity. Cuskelly et al. (3) explored the relationship between board composition, including the proportion of volunteers on the board, and organizational effectiveness in non-profit sports organizations. The study found that sports organizations with a higher proportion of volunteers on their board had higher levels of organizational effectiveness, as measured by factors such as goal attainment and stakeholder satisfaction. The financial analysis in the European project EVIS (30) showed that the total cost for the sports clubs to perform their current operations, would increase by 53%, across eight European countries (the ones examined here), if the clubs are not supported by volunteers.

Despite the importance of volunteering in sports, assigning a monetary value to this activity can be challenging. As mentioned before, traditional approaches to valuing volunteering focus on the input of individuals rather than the outcomes in terms of obtained benefits. For example, Taylor et al. (31) conducted a study that estimated the value of volunteering in sports clubs in England using the replacement cost approach. This approach values volunteering based on the cost of hiring paid workers to perform the same tasks as volunteers. The study found that the value of volunteering in sports clubs in England was approximately £14 billion per year. A Council of Europe study by Andreff et al. (32) calculated the economic significance of voluntary work via a replacement cost by wage rate of a sports teacher. They found that voluntary work was ranging between 0.03% (Sweden) and 2.2% (Spain) of the national Gross Domestic Product (GDP). Also, Vos et al. (33) showed by calculating the replacement cost that volunteers are a substantial resource for Flemish sports clubs and have a considerable economic value. Taking their market share into account, the Flemish sports clubs outnumber the fitness industry with regard to the economic value of human resources, but fitness and health clubs were found to be more cost-efficient. In a more recent study, Orlowski and Wicker (27) employed a similar methodology, relying on volunteers’ self-assessment of a reasonable wage for their specific activities. When applied to German sports clubs, they determined a monetary value of €9.98 for each hour of voluntary work.

These studies suggest that traditional approaches to valuing volunteering, such as the opportunity cost approach or the replacement cost approach, can provide useful estimates of the value of volunteering in sports based on the input of individuals. However, these approaches could be problematic because they are based on the labour market characteristics of the volunteers, or the activities provided by them (21). In this context, some studies have developed the contingent valuation model using an output-based perspective. For example, Orlowski and Wicker (21) obtained an interval of €17.51-€61.26 on one hour of voluntary work among German volunteers in sports clubs. However, it is important to note that these approaches may not fully capture the value of volunteering to individuals, as they do not consider the intangible benefits that individuals may derive from their participation. Most studies have focused on examining the influence on outcomes such as social capital and personal development (34), with the impact on well-being typically receiving less attention, as noted by Wicker & Downward (28).

### 2.2 The wellbeing valuation approach (WVA) for volunteering in sport

Research has revealed a positive and significant relation between sport volunteerism and SWB. Some studies have focused on the impact produced by sport volunteering. Lu et al. (35) showed a positive relationship between sport volunteerism and wellbeing among college students in Taiwan. Wicker and Downward (28) identified a positive association between wellbeing and sport volunteers in Europe, distinguishing between various voluntary roles. The positive relation is primarily observed in operational roles undertaken by volunteers, while other roles exhibit a negative association. Thormann, Gehrmann and Wicker (36) found that for German sports volunteers, the number of volunteering hours is positively linked to various aspects of SWB across different life domains. In a more recent study, Wicker, Thormann and Davies (37) examined various voluntary roles and different measures of SWB among football volunteers.

To address the challenges associated with valuing volunteering, researchers have begun to apply the WVA to this activity. This approach estimates the contribution of volunteering to individuals’ SWB and assigns monetary values by estimating compensation payments. In other words, as mentioned before, it calculates how much income individuals would be willing to forego in order to continue volunteering while retaining their current level of SWB.

A growing body of research has applied the WVA to estimate the monetary value of sports participation and physical activity. These studies have used data from large-scale surveys and advanced statistical methods to explore the relationship between volunteering and SWB and assign a monetary value to this activity. The review of Schoemaker (26) identified two potential biases that can arise when using the wellbeing valuation approach. One important bias is the endogeneity of income, which refers to the possibility that unobserved factors may influence both income and wellbeing, leading to biased estimates of the effect of income on wellbeing. To address this issue, some studies have used instrumental variable approaches or control function methods to account for unobserved heterogeneity and obtain unbiased estimates of the effect of volunteering on SWB. For example, Fujiwara, Kudrna and Dolan (25) exploited the randomness of lottery wins as an instrument and Lemyre, Mader and Ambard (38) used spouse income as an instrument. Both studies found that the income coefficient was much larger than predicted in standard linear regression. As mentioned in the (25) Kudma and Dolan (25) study, this is a generalised result. The instrumentalization of income would almost certainly increase the coefficient of income (β1), presented later on in the income compensation equation, compared to a standard linear model, leading to lower values for income compensation. Fujiwara, Kudrna and Dolan (25) used the control function approach in a 3-stage wellbeing model to make sure the estimations for the effect of income and the effect of leisure participation on SWB were unbiased. The control function allows one to derive estimates of the sample average partial effect (APE) for income for anyone in the sample, instead of the local average treatment effect (LATE), for only a subsample, resulting from a standard instrumental approach.

Another potential bias is the selection bias, which refers to the possibility that individuals who choose to participate, for example in physical activity or volunteering, may have different characteristics than those who do not, leading to biased estimates of the effect of the particular activity on SWB. To address this issue, some studies have used panel first-difference designs to control for observable differences between participants and non-participants and obtain unbiased estimates of the effect of participating on SWB. Overall, these potential biases highlight the importance of carefully designing studies and using appropriate statistical methods when applying the WVA to estimate the value of, in this case, volunteering in sports.

While the WVA has been applied to various non-market goods and services, there are few studies that have specifically applied this approach to value volunteering in sports. Thormann et al. (36) found that volunteering hours had a positive impact on satisfaction in different life domains, with the exception of work satisfaction. Thormann et al. (36) also found that the monetary values assigned to volunteering hours varied depending on the type of life domains under study (life, health, work, income and leisure satisfactions). The monetary value in terms of life satisfaction is estimated between €14 and €21 per hour, while in terms of leisure satisfaction values are higher (€46–47 per hour). Wicker et al. (37) obtained different monetised values of football volunteers depending on the measure of SWB applied and the voluntary roles played by the volunteers. In Europe, sport volunteers have been found to have a significant amount of monetised wellbeing, reaching the amount of 2,250 euros annually per volunteer (30).

In the United Kingdom, Lawton et al. (39) used first-difference estimation within the British Household Panel Survey and Understanding Society longitudinal panel datasets to control for higher prior levels of wellbeing of those who volunteer. They produce robust quasi-causal estimates by ensuring that volunteering is associated not just with a higher SWB *a priori*, but with a positive change in wellbeing. The study finds that volunteering is associated with an increase in SWB, with an equivalent value of £911 per volunteer per year on average.

Our current understanding of the value of sport volunteering, and more broadly volunteering in general, has been significantly advanced through the application of the WVA. This innovative technique enables researchers to assign a monetary value to the SWB improvements associated with volunteering, thus providing a tangible measure for an otherwise intangible benefit. Current research, such as the works of Lawton (39), Thormann et al. (36) and Wicker et al. (37) offers valuable insights into this realm, revealing promising yet complex relationships between volunteering and individual wellbeing. A complication in the model is that we reasonably assume that the point of action (volunteering) would impact on wellbeing. However, it can also be argued that this will create a loop where a rise in wellbeing will encourage more action, appearing as if the impact has the reverse direction. From the seminal work of Taylor et al. (10), different empirical studies have tried to establish the main determinants of sports volunteering, paying attention to the economy. These studies have shown a positive effect of income and education on sports volunteering [e.g., (12, 14, 40, 41)]. As Hallmann and Muñiz Artime (14) argue, volunteering, in general, is more affordable for those people with high levels of income. This positive effect seems to be more important than the higher opportunity costs of volunteering for this social group (42). At the same time, some studies have considered the role played by motivation in terms of decision and time of volunteering in sports, but there is no extensive empirical evidence about the direction of the relationship between volunteering and subjective well-being. One exception is Soukiazis and Ramos's paper (2016), where they concluded that volunteering activities, in general, have a positive and significant effect on life satisfaction, a proxy of subjective well-being. Considering this previous evidence, our theoretical framework considers that the way around the mechanism is from socio-economic position to volunteering and from volunteering to subjective well-being. This framework has been justified with our methodological approach based on instrumental variables. Further, this methodology has been commonly applied to establish the causality between sports participation and subjective well-being [e.g., (43)].

However, it is clear that more research is required to elucidate the full extent and nuances of these relationships. The current body of knowledge, although growing, remains limited, particularly concerning sport volunteering. This review has shown that there is a gap in the literature about the monetization of the intangible benefits derived from volunteering in sports. This gap has three main characteristics. Firstly, the monetization of volunteering activities in sports has not received deserved attention. Secondly, few studies have applied the WVA to estimate these benefits for a large set of individuals in different countries. Thirdly, endogeneity problems have become a challenge to overcome. This paper attempts to fill this gap by showing a methodological approach and empirical evidence about this monetization. In particular the potential biases, such as endogeneity and selection bias, identified in Schoemaker's review (2023), further underline the importance of the study design and statistical approaches in future investigations. By continuing to refine the methodologies and address these challenges, this study works towards unbiased, robust estimates of the effects of volunteering on SWB. This understanding is critical not only for recognising the full societal value of volunteering but also for shaping policies and strategies that encourage and support such socially beneficial activities.

## 3 Materials and methods

In this study a cross-sectional design was employed, collecting data from eight selected European countries. Cross-sectional studies, as records of a population at a specific point in time, are advantageous when studying the current status of a broad demographic. This method offers a robust approach to observe the present situation, making it an appropriate design for this study which aims to establish the value of sports volunteering in terms of SWB. The eight countries considered in this research are the ones that participated in EVIS project: Austria, Croatia, Germany, Greece, Lithuania, the Netherlands, Spain, and the United Kingdom.

### 3.1 Survey and variables

In order to estimate the value of volunteering in sport, a population survey in the aforementioned eight European states in 2022 was conducted (30). The objective is to identify the amount of SWB and social capital associated (or caused by volunteering). For this we assume that volunteering gives a person certain characteristics and social interactions that produce the wellbeing outcomes. To evaluate these outcomes, one needs to compare them not with the average case, where volunteers would be diluted within the general population, but with their absence, that is the case of non sport volunteers. Because of that two types of people were considered for the interview: volunteers in a sports club and the general population (other than sport volunteers). The first group of sport volunteers was screened out of nation-wide representative samples and the interviews were conducted on the phone. People, who defined themselves as being active as volunteers in a sports club were part of this target group. In the second group of general population, the interviews were also conducted by phone, screened out of a nation-wide representative sample. To differentiate from group 1, this group excluded sport volunteers. This exclusion helped in the monetisation of the wellbeing benefits.

Within the two target groups above, a total number of 1,091 people were questioned using telephone interviews. The biggest percentage of interviewees came from Germany (20.3 per cent) and the least from Netherlands (9.3 per cent). Since the focus was the study of sport volunteers compared to non-sport volunteers, the sample was split roughly in half consisting of 618 answers from the former group and 473 from the latter. The sample was random in terms of volunteers and in terms of non-sport volunteers but not overall for the population, serving to better estimate the monetisation of SWB associated with sport volunteering. Consequently, it should be clarified that the objective was not to obtain a representative panel for the entire country under investigation. The questionnaire was constructed based on standard socio-demographic questions and SWB measurement in terms of life satisfaction. The questionnaire was pretested through the Sheffield Hallam University online facility, and suggestions were implemented to develop the final questionnaire (30). The final survey was conducted by SpEA, from Austria, who can provide further data information upon request.

The most import variable in our questionnaire is the measurement of SWB, which is constructed via self-evaluation, with answers ranging from 0 to 10, for the whole sample, not just sport volunteers. Variables that have a relationship with SWB were included as well, such as work status (fulltime, home duties, student, unemployed or retired) and the composition of the household (number of adults/children), following previous empirical evidence about SWB determinants [i.e., (44–46)]. Self-assessed health status is measured on the scale from 1 (very poor) to 4 (very good). Income was identified with the question: What is the combined net monthly income of your household? To avoid exact identification, answers could be classified in 12 categories with a range of 1,000 euro (e.g., 2,000–3,000 euros). All the monetary values were eventually transferred into euros.

Volunteering was measured by the number of hours and minutes of volunteering during the past four weeks. When looking at the sample, an additional restriction to avoid outliers in the volunteering time was included. A restriction of a maximum number of hours of volunteering of 20 h per week was considered. Since most volunteers in sports may be sport participants or club members, it was also important to control for sport participation and club membership in the evaluated models. Further, to control for endogeneity in the case of income and volunteering, two instrumental variables were included. The first was the membership of a trade union and the second if the parents had done volunteering at any time. These instruments are explained in Section 3.3.

### 3.2 Data analysis and model

All statistical analysis was conducted using SPSS. At the first level of analysis, the survey overall had no missing values except in the case of income where around 10 per cent of the sample was missing. This is generally very little. However, because of the size of the sample the decision was taken to ‘fill’ the missing values using a means analysis by country and demographic characteristics. Given the very detailed framework of analysis no additional clustering was created in the data. The last stage in the income processing was to attach a monetary value to each income class, for example instead of the class 1,000–<2,000, the value 1,500 euros was used. In all cases it was ensured that the variables had a consistent data presentation and with consistent logic across the dataset. Minutes of volunteering was limited to 5,000 min in the past four weeks (20 h per week).

The WVA comprises three key stages: establishing the impact of income on SWB, determining the impact of a non-market good (in this case, volunteering) on SWB, and using the relationship between income and SWB to attribute a monetary value to the non-market good. This method allows for a comprehensive understanding of the monetary value of sport volunteering and its relationship to SWB, facilitating a broader appreciation of the societal benefits that such activities offer.

The equation used for estimating the effect of sport volunteering on SWB is based on previous empirical work [(25)]. The theoretical framework is centred on the estimation of the equations that aim to estimate the coefficients β_1_ and β_2_ in the income compensation formula (1):

(1) $$IC=M-e^{\left(\ln(M)-\frac{\beta_{2}}{\beta_{1}}\right)}$$

Where β_1_ refers to relationships between income and SWB and β_2_ refer to a relationship between SWB and sport volunteering; M represents the average annual disposable income, of an individual, in each case considered. Because of data restrictions the estimation can only take place for the set of eight countries as a whole. Then by changing the value of average personal income (M) estimations can be derived for each individual country. That means, that for practical reasons, the estimated values of β_1_ and β_2_ are assumed to be applicable in all the countries under consideration.

### 3.3 Causal effects and instrumental variables

On the one hand, understanding the causal relationship between income and SWB is central to the WVA. This relationship informs the estimation of how income changes impact an individual's overall SWB. There is large empirical evidence about positive associations between income and SWB (i.e., (45, 46). To accurately evaluate this causal relationship, econometric models accounting for various socio-demographic factors like age, gender, education, and work status was employed. These models isolate the specific impact of income on SWB, ensuring that the relationships observed are not confounded by these other variables. Addressing potential endogeneity issues, which emerge when income and SWB simultaneously influence each other, or by unobservables, was another crucial step in the methodological approach. An instrumental variable technique was used to isolate the causal impact of income on wellbeing.

In this article, membership of a trade union has been selected as the instrumental variable. The theoretical grounding for this choice lies in the recognised economic and social role of trade unions. Traditionally, trade unions are entities devoted to negotiating better wage and employment conditions for their members, thereby having a significant impact on members’ income levels (47). This characteristic of trade unions forms the basis for their selection as an instrumental variable in the analysis. The key presumption here is that the relationship between trade union membership and an individual's life satisfaction is mediated primarily through its effect on income, thus forming an indirect link between the instrumental variable and the dependent variable.

In more precise terms, while trade union membership can lead to enhanced income levels due to better employment terms, its direct impact on life satisfaction remains weak. This notion is substantiated by empirical data, which exhibits only a marginal direct relationship between trade union membership and wellbeing, with a correlation coefficient of 0.01 and a *t*-value of less than 0.001. The weak association suggests that any notable effect of trade union membership on life satisfaction is likely channelled through its influence on income, thus fulfilling the instrumental variable assumptions of relevance and exclusion.

On the contrary, the data reveals a more pronounced direct relationship between trade union membership and income, as evidenced by a correlation coefficient of 0.14 and a *t*-value of less than 0.001. This relationship validates the strong association between the instrumental variable and the explanatory variable (income), which is a critical assumption for the application of the instrumental variable method.

Further empirical validation of the instrument choice comes from the significant *T*-value of 3.0 and F-statistic of 73 in the model. These statistical measures underscore the strength and relevance of trade union membership as an instrumental variable in this setting. The high F-statistic, in particular, is indicative of the strong explanatory power of the instrument, thereby reducing the likelihood of weak instrument bias—a common pitfall in instrumental variable analysis. In the two-stage econometric model, consistent with Fujiwara, Kudrna and Dolan (25), demographic background factors and country variables were included in the first stage regression. Subsequently, the residuals from this first stage regression in the second stage to correct for endogeneity and unobservables were used. This two-step approach allowed to rigorously address potential biases from omitted variables and endogeneity, ensuring the integrity of the estimates.

On the other hand, establishing the causal impact of volunteering on wellbeing forms the other pivotal equation in the WVA framework. This relationship determines the non-market value that volunteering activities confer in terms of enhancing an individuals’ SWB. To investigate this relationship, again an instrumental variable approach is applied, much like the previous methodology for assessing income's effect on wellbeing.

The selection of the instrumental variable stems from the well-documented sociological premise that parental behaviours can significantly shape the behavioural tendencies of their offspring, including their inclination towards volunteering (48). In this light, the historical volunteering status of an individual's parents was adopted as the instrument. This choice is grounded in the hypothesis that while parental volunteering may foster a propensity for volunteering in the individual, it is less likely to have a direct bearing on an individual's life satisfaction. Aydinli et al. (49) further supported this by showing that the motivational structure for volunteering remained consistent across different contexts, indicating a learned behaviour rather than an inherited trait influencing volunteering. However, recognising that other inherited or environmentally induced characteristics could confound this relationship, the regression have controlled for age, gender, and education in the analysis to mitigate potential biases. Previous evidence in sport participation have considered parental participation to explain individual involvement in sports (50).

Our data analysis lends support to this conceptual framework. The findings revealed that parental volunteering bears a modest relationship with the wellbeing of the respondents, as indicated by a correlation coefficient of 0.13 and a *t*-value of less than 0.001. More significantly, a larger and significant relationship emerged between parental volunteering and the extent of volunteering by the respondents, as measured by minutes volunteered, with a correlation coefficient of 0.33 and a *t*-value of less than 0.001. This highlights a robust association between the instrumental variable and the explanatory variable (volunteering), thus satisfying the relevance condition of the instrumental variable approach.

Further empirical validation of the instrument choice is gleaned from the noteworthy *T*-value of 8.2 and F-statistic of 42 in the model. These statistical measures accentuate the strength and relevance of using parental volunteering as an instrumental variable in this scenario. The high F-statistic, in particular, is indicative of the strong explanatory power of the instrument, which significantly mitigates the concerns surrounding weak instrument bias—a common issue in instrumental variable analysis.

Similar to the analysis of the causal effect of income on wellbeing, demographic background factors and country variables are incorporated in the first stage of the regression. The residuals from this first stage were then employed in the second stage to correct for any potential bias due to unobservables and endogeneity. By using parental volunteering as an instrument, this study was able to isolate the specific impact of volunteering on SWB, ensuring that the relationships observed were not influenced by other confounding variables. This strategy allowed the analysis to isolate the effect of volunteering from the potential effects of shared family background or characteristics that might also influence SWB.

In this research, monotonicity of the instrumental variable-exposure association is assumed, alongside linearity and homogeneity of the exposure-outcome relationship. As the results show later on, it can be concluded that volunteering has a causal effect on well-being, although no definitive conclusion about the direction of this effect is possible, as outlined by Burgess et al. (51). Recognizing the limitations of a cross-sectional study compared to longitudinal studies, efforts were made to mitigate the potential for reversed causality through the use of instrumental variables. Support for this approach is found in the studies of Binder (52) and Lawton (39), which utilize panel data to gain more robust insights into the causal relationship between volunteering and SWB. Binder's study provides an advanced analysis by examining the heterogeneity in the impact of volunteering on SWB, taking into account the existing level of well-being of volunteers. Lawton's research further strengthens the causal interpretation by employing first difference regressions alongside fixed effects, thus isolating prior happiness trends to derive quasi-causal estimates of the impact of volunteering. The findings suggest a causal relationship, although the definitive direction of this effect remains open to further investigation.

## 4 Results

### 4.1 Descriptive analysis

The estimation of the SWB index follows the principles outlined before and is presented in Table 1. The SWB values have a hypothetical maximum value of 10 and relate to all the people in the sample, not just the sport volunteers. According to Table 1, the mean value of SWB across the eight counties examined is 7.3, with median value in the sample at 7. The closeness of the two averages increases confidence in the sampling in terms of SWB. The maximum average value of SWB appears in Germany (7.7), followed by Spain (7.5) and Lithuania (7.4).

**Table 1:** Average value of subjective wellbeing (SWB) per country.

Country	Average SWB value
Austria	7.1
Croatia	7.3
Germany	7.7
Greece	6.8
Lithuania	7.4
Netherlands	7.4
Spain	7.5
United Kingdom	7.3
Total (average)	7.3
Total (median)	7

Further, it is of greater interest to examine the average difference of the SWB index between sport volunteers and non-sport volunteers. If any real effect on wellbeing can be demonstrated to exist, then at the very least the sport volunteers should have a higher SWB average index across the eight countries examined.

Table 2 shows the average SWB scores between sport volunteers and non-sport volunteers across the eight examined countries. The sport volunteers have an average score of 7.6 compared to no sport volunteers’ score of 7.0. In other words, the sample suggests that sport volunteers have 9 per cent more wellbeing than no sport volunteers. A statistical t test for the equality of the two means, rejected the null hypothesis at the 1 per cent level, giving confidence in the result.

**Table 2:** Average value of subjective wellbeing (SWB): volunteers.

	Average SWB value
Sport volunteers	7.6
No sport volunteers	7.0
*T test for equality of means*	Rejected at 1% level

Table 3 presents the descriptive statistics of 18 variables measured on a sample of 1,091 individuals from eight European countries. The sample is distributed between males (56%) and females (44%). 65% of the sample is working fulltime, 3% does home duties, 5% are students, 7% are unemployed and 9% are retired. One third of the sample is a member of a trade union and 40% has a parent who has done volunteering in the past. A large share of the sample has done moderate or vigorous sport/active recreation during the last 4 weeks and 62% is a member of a sports, health or fitness club/centre. Note that the sampling done ensured the inclusion of a large number of volunteers into the analysis.

**Table 3:** Descriptive statistics.

Variables	Measurement	*N*	Min.	Max.	Mean	S.D.
Gender	Binary	1,091	0	1	0.56	0.50
Working full time	Binary	1,091	0	1	0.65	0.48
Home duties	Binary	1,091	0	1	0.03	0.18
Student	Binary	1,091	0	1	0.05	0.21
Unemployed	Binary	1,091	0	1	0.07	0.25
Retired	Binary	1,091	0	1	0.09	0.28
Memberships of trade unions	Binary	1,091	0	1	0.33	0.47
To the best of your knowledge. has any of your parents volunteered in sport at any time?	Binary	1,091	0	1	0.40	0.49
Have you done any moderate or vigorous sport/active recreation during the last 4 weeks?	Binary	1,091	0	1	0.84	0.37
Are you a member of a sport, health or fitness club/centre?	Binary	1,091	0	1	0.62	0.49
Age over 16	Continuous	1,091	16	79	42	13
Number of adults in household	Continuous	1,091	1	6	2.09	0.94
Number of children in household	Continuous	1,091	0	5	0.65	0.85
Log of monthly income	Continuous	1,091	3.91	9.31	7.85	0.90
On a scale of 0 (not at all) to 10 (completely). Overall, how satisfied are you with your life in general (SWB)	Continuous	1,091	0	10	6.31	1.96
Minutes volunteering in sport during the past 4 weeks	Continuous	1,091	0	5.000	520	6.40
					Freq.	Percent
Education	Primary education				18	1.6
	Lower secondary education				52	4.8
	Higher secondary education				239	21.9
	Bachelor professional				237	21.7
	Bachelor academic				264	24.2
	Master academic				205	18.8
	Doctorate				76	7
How do you evaluate your own health?	Not good at all				49	4.5
	Not so good				205	18.8
	Good				475	43.5
	Very good				362	33.2

The average age is 42 years. The average household has 2.09 adults, 0.65 children and a monthly log of income of 7.31, corresponding to a value of 2,566 euro. For SWB index, the average value is 7.3, on a scale of 0–10. Note that volunteering in this sample does not reflect the level of engagement in any country, because half of the sample was, by design, selected because they volunteered in sports and they are no representatives of the whole population in each country.

### 4.2 Causal effect of income on wellbeing (β_1_)

The first stage of the regression models involved estimating the log of income using a comprehensive model that included demographic background variables to account for their potential influence on income. Additionally, a variable to correct for differences among countries based on their income levels was incorporated. This ensured that model 1 in Table 4 accurately reflected the varying economic conditions across the different European countries included in the study.

**Table 4:** Regression results for estimating the causal effect of income on wellbeing (SWB).

	Model 1	Model 2
	1st stage: Log of income	2nd stage: SWB with covariates
	B (SE)	B1 (SE)
Constant	6.19 (0.12)^c^	−4.31 (4.89)
Age. over 16	0.00 (0.00)	0.00 (0.01)
Males	0.19 (0.05)^c^	−0.12 (0.19)
Education	0.17 (0.02)^c^	−0.19 (0.14)
Country ranked according to income	0.14 (0.01)^c^	−0.13 (0.11)
Membership of trade unions	0.15 (0.05)^b^	
Log of monthly income		1.37 (0.79)^a^
Residual 1 stage		−2.45 (0.84)^b^
Residual^a^ log of income		0.17 (0.05)^c^
Number of adults in household		−0.06 (0.06)
Number of children in household		0.10 (0.07)
How do you evaluate your own health?		0.77 (0.07)^c^
Omitted group: Working fulltime		
Home duties		−0.03 (0.32)
Student		−0.29 (0.29)
Unemployed		−0.54 (0.23)^b^
Retired		0.16 (0.23)

^a^ Statistical significance at 10% level.

^b^ Statistical significance at 5% level.

^c^ Statistical significance at 1% level.

A key feature of this first-stage regression is the inclusion of the chosen instrument, membership of a trade union, which is found to be significant. This is critical because a significant instrument demonstrates its relevance in predicting changes in the variable of interest, in this case, the log of income. The residuals from this first-stage regression, a measure of the unexplained variance, were then collected. These residuals are important because they help to correct for potential biases due to unobservables in the second stage of the regression, as a control function. In model 2, estimating the effect on SWB, the log of income was found to be significant. This finding (β_1_) reveals that a 1% increase in income is associated with a 0.0137-point improvement in SWB. Given the similarity with previous evidence [i.e., (25)], this outcome offers a robust causal impact of income on SWB.

### 4.3 Causal effect of volunteering on wellbeing (β_2_)

The next component centred on estimating the causal effect of volunteering, specifically the number of minutes spent volunteering in the last four weeks, on SWB (β_2_). In the first stage of this process, a variable was leveraged asking whether the respondents’ parents had volunteered, employing it as an instrumental variable, and included country and demographic background variables (Model 3, Table 5). As mentioned before, this variable was selected because it is correlated with the respondent's volunteering behaviour but does not directly affect their wellbeing, making it a suitable instrument. Then the residuals from this first-stage regression were collected, which captured the unexplained variance not accounted for by the variables in the model. These residuals play an integral role in the next stage, as they allow to correct for any potential bias from unobservables and endogeneity.

**Table 5:** Regression results for estimating the causal effect of sport volunteering on wellbeing (SWB).

	Model 4	Model 5
	1st stage: minutes, sport volunteering	2nd stage: SWB with covariates
	B (SE)	B (SE)
(Constant)	640 (242)^c^	3.6 (0.6)^c^
Country ranked according to income	65 (20)^c^	0.0 (0.0)^b^
Males	−21 (4)^c^	−0.1 (0.1)
Age over 16	440 (94)^c^	0.0 (0.0)
Education	74 (33)^b^	0.0 (0.0)
Have your parents volunteered in sport at any time?	819 (100)^c^	
Sport volunteering minutes		0.0004 (0.0)^b^
Residual 1 stage		0.0 (0.0)
Residual × minutes volunteering		0.0 (0.0)
Number of adults (18 + years)		0.0 (0.0)
Number of children (		0.1 (0.1)
How do you evaluate your own health?		0.8 (0.8)^b^
log of monthly income		0.0 (0.0)
Omitted group: working fulltime		
Home duties		0.1 (0.1)
Student		−0.2 (0.2)
Unemployed		−0.4 (0.4)^a^
Retired		0.2 (0.2)
Have you done any moderate or vigorous sport/active recreation during the last 4 weeks? Moderate sport		0.2 (0.2)
Are you a member of a sport, health or fitness club/centre?		0.1 (0.1)

^a^ Statistical significance at 10% level.

^b^ Statistical significance at 5% level.

^c^ Statistical significance at 1% level.

In the second stage of the regression (Model 4 in Table 5), these residuals help to estimate the causal effect of the minutes spent volunteering on SWB. The results of this stage revealed a significant impact of a minute of volunteering on SWB at 0.0004. The additional variables account for other influences on SWB, helping to isolate the specific contribution of volunteering. The average sport volunteer had approximately 520 min of engagement in a four-week period. This translates into coefficient β_2_, as volunteering is contributing on average, to a 0.21-point improvement (approximately 520 × 0.0004) in SWB levels (measured from 0 to 10).

### 4.4 Monetary value of volunteering in sport

Table 6 estimates the income compensation of a sport volunteer assuming volunteering in the last four weeks. The official average income in the population per country was used and the values 0.21 for β_2_, and 1.37 for β_1_. What is important is that this monetisation of SWB, also includes the instrumentation of sport volunteering, increasing the confidence in the causality of the outcome. Using national annual income, the results were translated to reflect the purchasing power of each nation. This way, the monetary value of volunteering is widely distributed between €16 to €50 per hour, equivalent to between € 1,700 and € 5,200 per year, depending on the nationality of the volunteer.

**Table 6:** Monetisation of the generated SWB by sport volunteering.

		Stage 1	Stage 2	Stage 3	Value per hour volunteering
	Average annual income of the population	Causal effect of income on SWB (β_1_)	Causal effect of severe IV in sport on SWB (β_2_)	Income compensation	
Austria	€36,636	1.37	0.21	€ 5,200	€ 50.00
Croatia	€12,288	1.37	0.21	€ 1,700	€ 16.35
Germany	€35,316	1.37	0.21	€ 5,000	€ 48.08
Greece	€12,600	1.37	0.21	€ 1,800	€ 17.31
Lithuania	€13,392	1.37	0.21	€ 1,900	€ 18.27
The Netherlands	€34,008	1.37	0.21	€ 4,800	€ 46.15
Spain	€22,416	1.37	0.21	€ 3,186	€ 30.63
United Kingdom	€32,568	1.37	0.21	€ 4,600	€ 44.23
Average	€26,382	1.37	0.21	€ 3,700	€ 35.58

## 5 Discussion and conclusions

The monetary valuation of non-market goods and services is receiving a growing attention in the last years. Significant attention has been directed toward the amount of time dedicated to sport volunteering in Europe. This is particularly noteworthy as sports clubs play a central role in promoting both formal and informal participation. The sports club system is Europe is mainly based on the role played by volunteers (1, 2). However, a decrease of volunteers in sports may be observed over the last decade as indicated by European Commission's Special Eurobarometers on Sport and Physical Activity in the European Union (53–55). What was already a low rate of citizens engaged in sport volunteering in 2013 (7%), it declined further in 2017 (6%) and remained at that level in 2022 (6%). Volunteering in sports remains under-recognised for its social and economic benefits in Europe. Further decline of the rate of citizens engaged in volunteering in sports may be expected due to the lack of public policies and measures for the promotion of volunteering in sports in the European countries. Failure to implement policies for increasing voluntary engagement in sport organisations represents a missed opportunity to multiply benefits for both volunteers and participants in sport, and especially to attract the population that is not naturally inclined towards sport participation but could be attracted to the opportunity for self-actualisation through voluntary work in sport. Policies that combine measures to increase volunteering and participation could be considered. Public policies aimed at increasing the number of volunteers in sport organisations, especially in sport clubs in local communities, could have a multiple positive effect on sport and sport-related sectors. Increased number of volunteers could improve not only the wellbeing of volunteers but also increase the number and scope of services offered by sport clubs to local population, thus increasing participation and associated benefits related to participants’ health, social inclusion, etc. However, public policies targeting increased participation in sport seldom include actions to increase voluntary work.

Despite the importance of volunteering in sports, assigning a monetary value to this activity can be challenging. Traditional approaches to valuing volunteering (i.e., replacement and opportunity cost approaches), focus on the input of individuals rather than the outcomes in terms of obtained benefits (18, 20, 21). These approaches may not fully capture the value of volunteering to individuals, as they do not consider the intangible benefits that individuals may derive from their participation.

In this context, this research provides added value to the monetisation of volunteering in sports by applying the wellbeing valuation approach for the first time to a cross-sectional data in eight European countries. This model is based on the minutes of volunteering in sports and uses instruments both on the income and volunteering variables. This allows to estimate the casual impact of volunteering in sports more accurately and to assign reasonable monetary value to this activity. The panel data studies of Binder (52) and Lawton et al. (39) offer additional support. These studies employ panel data and therefore provide robust insights into the causal relationship between volunteering and SWB. These studies, particularly in their exploration of heterogeneity and prior happiness trends, reinforce the causal interpretations presented in this research.

The results show an average annual income compensation for a sport volunteer of €35.58 per hour devoted to volunteering in sport, resulting in a yearly compensation of € 3,700. There are large differences between the European countries because of different annual incomes. However, the sport volunteers in their actions, receive a value of annual wellbeing equivalent to approximately a monthly wage payment. These figures should not be considered as the monetary value that the volunteers would be prepared to pay for their engagement but a monetary value equivalent to the wellbeing they receive. The results of this study are similar to the ones obtained in the EVIS project, which applied a similar methodology (30). They estimated an equivalent of approximately €2,250 per year as the monetary value of sport volunteers in Europe in terms of SWB.

Although the valuation of income compensation of volunteering differs between countries, the study average (€ 35.58) and country specific outcomes (€ 48.08 for Germany) are in line with the outcomes of the study by Orlowski and Wicker (27), who found an interval of € 17.51—€ 61.26 based on a contingent valuation method. As mentioned, the current approach is wider, because it includes intangible benefits of volunteering. The current outcomes also show that the total valuation based on the current approach is much higher than the direct self-assessed wage that (German) volunteers determined for each hour of voluntary work (€ 9.98 in 2015). This also highlights the size of the intangible effects of volunteering for an individual's wellbeing. Contrasting the results of this study with Thormann et al.'s (36) values, the average European estimates are higher for life satisfaction values, but lower than leisure satisfaction values. In addition, the findings of the current study and that of Lawton et al. (39) manifest a compelling narrative on the positive association between volunteering and subjective wellbeing. While Lawton et al.'s methodology provides a strong design by accounting for individuals’ initial wellbeing levels, it might potentially lead to an underestimation of the true causal effect if the impact of volunteering accumulates over time rather than manifesting immediately. The First Difference methodology would capture the immediate changes in wellbeing but might miss out on the longer-term accumulative effects. In contrast, the instrumental variable approach in this study, if the instruments are well-chosen and the exclusion restriction holds, could potentially capture a broader causal effect over a longer time horizon, explaining the higher estimated income compensation value for volunteering.

### 5.1 Practical implications for decision makers

The fact that volunteering generates a double dividend of increasing the social participation rate in sport as well as the individual well-being of the volunteers, should be no argument for lowering the public support of sport because of citizens’ self-realization. The results at hand are only possible in a society which recognises and appreciates the positive external effects of volunteering in sports, and where necessary sport infrastructural facilities are also available.

From the results of this study some policy implications could be argued. Our research shows that volunteering in sport may incorporate substantial monetary value associated with increased wellbeing. Therefore, public policies promoting volunteering in sports may be beneficial for both a society's and individual's wellbeing as well as the sports sector. One avenue for policy intervention is to introduce financial incentives, such as individual tax deductions or credits, which have been introduced in the Netherlands and several other countries (30, 56, 25, 57–60). Another critical area for policy attention is the provision of training and resources. By organizing free training sessions or workshops, volunteers can be equipped with essential skills that elevate the quality and impact of their service. Moreover, public recognition can significantly boost volunteer morale and motivation. Annual awards or acknowledgment ceremonies for outstanding volunteers can underscore society's value for these unsung heroes. This sentiment can be further amplified by introducing or bolstering volunteer programs in educational institutions, integrating them into curricula as credit-based initiatives. Further, ensuring that volunteer-reliant organizations, like sports clubs, receive sufficient support is paramount. By offering grant programs or funding support, governments can safeguard the sustainability and expansion of these critical entities and their specific infrastructures. The dissemination of good practices among countries, organizations and people is also important. It is essential to connect people of different ages, genders, traditions, attitudes and values for meaningful volunteering.

For stakeholders like individuals, sports clubs, and associations, these findings can drive a renewed emphasis on valuing volunteers. Recognising the role of volunteers is a win-win situation: it not only benefits the individual volunteer but also fosters a supportive community and offers tangible cost-saving advantages to the club they serve. Additionally, as the sports sector undergoes increasing professionalization, a comprehensive understanding of the interplay between voluntary contributions and professional roles can inform future strategies and policies.

### 5.2 Limitations and further research

The design of this study notwithstanding, there are several limitations that need to be acknowledged. Firstly, the instrumental variables chosen—membership in a trade union and parental volunteering—while theoretically grounded, may carry unobserved confounders that could potentially bias the estimates. Although the study controlled for age, gender, and education in the analysis to mitigate such biases, the extent to which these instruments are exogenous to individual wellbeing remains a topic for further discussion and verification. Secondly, the sample exhibited a significant over-representation of volunteers, constituting half of the sample. Since the basis of the analysis comes from a comparison between volunteers and non-volunteers, the biggest threat comes from any underrepresentation of volunteers. The methodology used ensured that we have sufficient information on volunteers at the cost of not being able to establish the rates of engagement, which were taken from other surveys, such as the Eurobarometer. This demographic skewness likely impacts upon the extension of the findings to the broader population, as the sample does not mirror the volunteering rate in the general populace. The oversampling of volunteers may have introduced a selection bias which, in turn, could have influenced the observed relationships between volunteering, income, and wellbeing. For example, it is posited that for individuals with higher incomes, a change in income exerts a smaller impact on SWB compared to those with lower incomes. Expanding the sample to include volunteers, who typically have higher incomes, could depress the coefficient, thereby elevating the monetary value. Moreover, the reliance on self-reported measures for wellbeing and volunteering activities may engender response biases, such as social desirability or recall bias. The accuracy and reliability of such self-reported metrics are contingent on the honesty and self-awareness of the respondents, factors that are inherently difficult to ascertain or control for. Lastly, the cross-sectional nature of the dataset precludes definitive conclusions regarding the temporal dynamics between income, volunteering, and wellbeing. The causal pathways elucidated through this study provide a snapshot within a specific timeframe but do not capture potential lagged effects or feedback loops over time.

At the same time, further research questions appear to be afforded in the future. Other studies, in sport participation, where wellbeing effects are present, point out to the possibility that sport volunteering may lead to mental health benefits, with savings for the national health systems, positive educational effects leading to gains in productivity, higher incomes, and less crime. Further research is required in these directions. The monetisation of the possible social benefits followed from the rises in SWB, should be sufficient to steer future research in this direction. Also, the inclusion of different life domains for measuring SWB and the distinction into several voluntary roles, as previous evidence has done, with an expansion of the sample size per country would allow to obtain better monetary values. In future research, it may be valuable to consider cultural values, traditions, and evolving perspectives on volunteering, which can vary significantly between countries. In addition, analysis can be designed to compare the population of non volunteers with volunteers of any kind before introducing sport volunteering on top of that in order to identify if there is any special advantage of sport volunteers compared to volunteering in general. Such considerations could help us better understand their potential impact on SWB and the process of assigning a monetary value to it.

## Data Availability

The wellbeing dataset presented in this article is not readily available; it is held by SpEA, Austria, which must be contacted for this purpose. Any application to access the wellbeing dataset must be directed to Guenther Grohall at ‘guenther.grohall@spea.at’. Further enquiries can be directed to the corresponding author.
